# Supplementary material for: Saccharomyces boulardii (CNCM I-745) improves intestinal damage in sepsis by remodeling intestinal flora structure
Source: Front Cell Infect Microbiol. 2025 Jul 31;15:1602792. doi: 10.3389/fcimb.2025.1602792 (PMC12350329; doi:10.3389/fcimb.2025.1602792)
Supplement: Supplementary file 1 [file DataSheet1.zip › summary/summary/7_Differential_analysis/3_Indicator_result/4_Order/Order_indicator_Group.pdf]

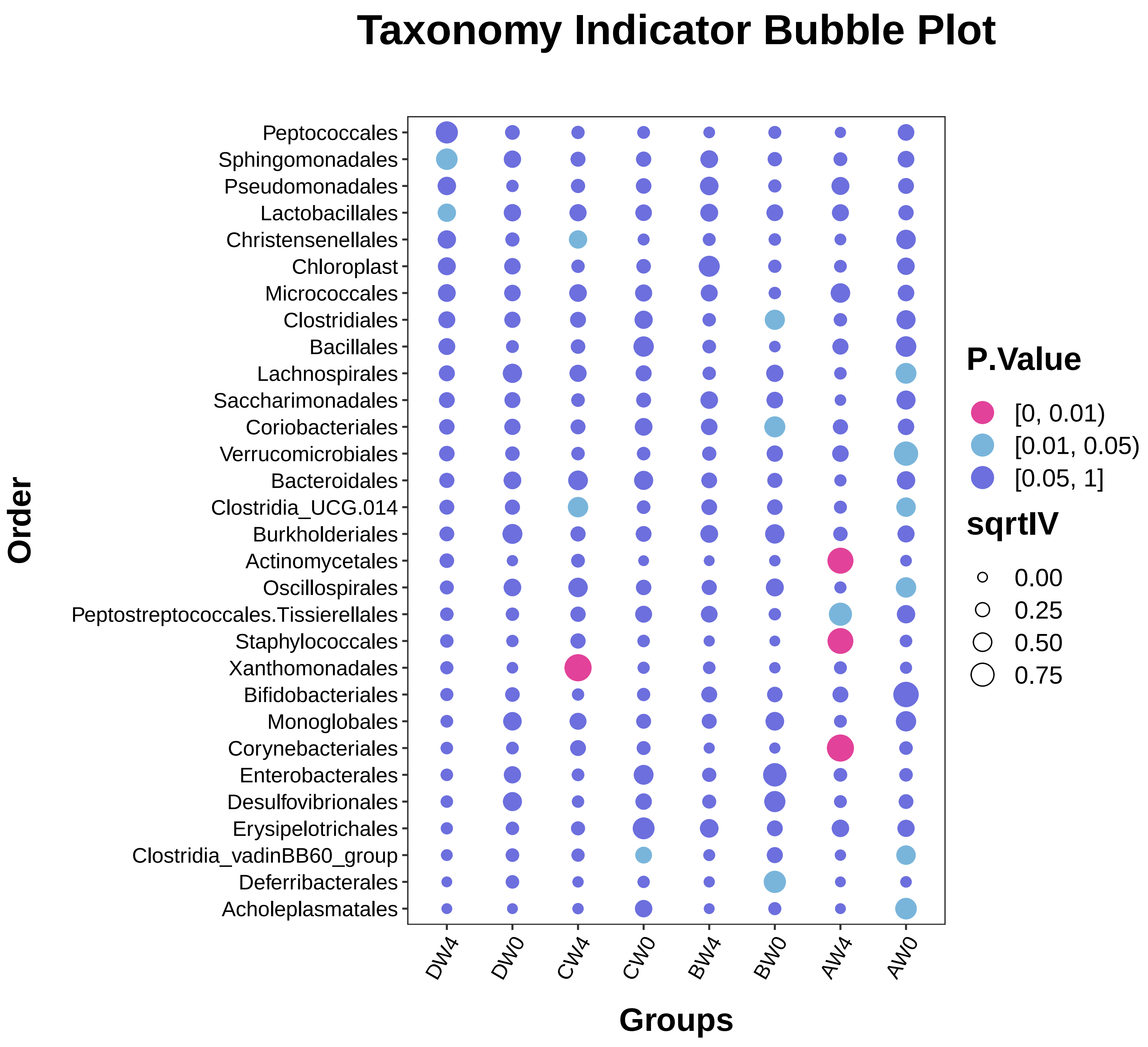
Taxonomy Indicator Bubble Plot
Order
Peptococcales
Sphingomonadales
Pseudomonadales
Lactobacillales
Christensenellales
Chloroplast
Micrococcales
Clostridiales
Bacillales
Lachnospirales
Saccharimonadales
Coriobacteriales
Verrucomicrobiales
Bacteroidales
Clostridia_UCG.014
Burkholderiales
Actinomycetales
Oscillospirales
Peptostreptococcales.Tissierellales
Staphylococcales
Xanthomonadales
Bifidobacteriales
Monoglobales
Corynebacteriales
Enterobacterales
Desulfovibrionales
Erysipelotrichales
Clostridia_vadinBB60_group
Deferribacterales
Acholeplasmatales
DW4
DW0
CW4
CW0
BW4
BW0
AW4
AW0
Groups
P.Value
[0, 0.01)
[0.01, 0.05)
[0.05, 1]
sqrtIV
0.00
0.25
0.50
0.75
